# Protocol: simulation training to improve 9-1-1 dispatcher identification of cardiac arrest

**DOI:** 10.1186/s12873-016-0073-6

**Published:** 2016-02-01

**Authors:** Hendrika Meischke, Ian Painter, Anne M. Turner, Marcia R. Weaver, Carol E. Fahrenbruch, Brooke R. Ike, Scott Stangenes

**Affiliations:** University of Washington, Northwest Center for Public Health Practice, 1107 NE 45th St. Suite 400, Seattle, WA 98105 USA; University of Washington, Institute for Health Metrics and Evaluation, 2301 Fifth Ave, Room 436, Seattle, WA 98121 USA; Public Health- Seattle and King County, Division of Emergency Medical Services, 401 5th Ave Suite 1200, Seattle, WA 98104 USA; Department of Family Medicine, University of Washington, 4225 Roosevelt Way NE, Suite 308, Seattle, WA 98105 USA

**Keywords:** Resuscitation, Cardiac arrest, 9-1-1 dispatcher, Emergency medical services, Cardiopulmonary resuscitation, Simulation, Standardized patients, T-CPR

## Abstract

**Background:**

9-1-1 dispatchers are often the first contact for bystanders witnessing an out-of-hospital cardiac arrest. In the time before Emergency Medical Services arrives, dispatcher identification of the need for, and provision of Telephone-CPR (T-CPR) can improve survival. Our study aims to evaluate the use of phone-based standardized patient simulation training to improve identification of the need for T-CPR and shorten time to start of T-CPR instructions.

**Methods/Design:**

The STAT-911 study is a randomized controlled trial. We will recruit 160 dispatchers from 9-1-1 call-centers in the Pacific Northwest; they are randomized to an intervention or control group. Intervention participants complete four telephone simulation training sessions over 6–8 months. Training sessions consist of three mock 9-1-1 calls, with a standardized patient playing a caller witnessing a medical emergency. After the mock calls, an instructor who has been listening in and scoring the dispatcher’s call management, connects to the dispatcher and provides feedback on select call processing skills. After the last training session, all participants complete the simulation test: a call session that includes two mock 9-1-1 calls of medium complexity. During the study, audio from all *actual* cardiac arrest calls handled by the dispatchers will be collected. All dispatchers complete a baseline survey, and after the intervention, a follow-up survey to measure confidence. Primary outcomes are proportion of calls where dispatchers identify the need for T-CPR, and time to start of T-CPR, assessed by comparing performance on two calls in the simulation test. Secondary outcomes are proportion of actual cardiac arrest calls in which dispatchers identify the need for T-CPR and time to start of T-CPR; performance on call-taking skills during the simulation test; self-reported confidence in the baseline and follow-up surveys; and calculated costs of the intervention training sessions and projected costs for field implementation of training sessions.

**Discussion:**

The STAT-911 study will evaluate if over-the-phone simulation training with standardized patients can improve 9-1-1 dispatchers’ ability identify the need for, and promptly begin T-CPR. Furthermore, it will advance knowledge on the effectiveness of simulation training for health services phone-operators interacting with clients, patients, or bystanders in diagnosis, triage, and treatment decisions.

**Trial registration:**

ClinicalTrials.gov Registration Number: NCT01972087. Registered 23 October 2013.

## Background

An estimated 326,200 adults suffer an out-of-hospital cardiac arrest (OHCA) each year in the United States, and a patient’s chance of survival decreases with every minute that they do not receive cardiopulmonary resuscitation (CPR) [1–3]. For patients in Emergency Medical Services (EMS) assessed cardiac arrest, time from 9-1-1 call start to Advanced Life Support (ALS) arrival may be as long as seven minutes, suggesting that the most critical time for interventions that increase survival may be before EMS arrives [4]. For a witnessed collapse, bystander CPR is an effective intervention in that time before EMS arrives [5]. To increase bystander CPR, 9-1-1 dispatchers give CPR instructions to callers over the phone [6, 7], Telephone-CPR (T-CPR), which has significantly increased survival from cardiac arrest [8]. In cases where dispatchers do not recognize the need for T-CPR and do not provide T-CPR instructions to the caller, survival decreases [6, 9]. Given the low survival rates for out-of-hospital cardiac arrest in most communities [1, 4, 5], increasing dispatcher identification of the need for T-CPR and expediting initiation of T-CPR could have a meaningful effect on population health nationwide.

To any one 9-1-1 dispatcher, cardiac arrest calls are infrequent, yet it is critical that they handle these incidents effectively. While dispatchers cannot definitively *diagnose* cardiac arrest over the phone, they are instructed to begin T-CPR if a caller reports on a patient who is *not* conscious and *not* breathing *normally.* Typically, consciousness and breathing questions are asked by dispatchers during an “all-caller protocol,” a scripted set of introductory questions asked of all medical emergency callers to help determine the appropriate EMS response and pre-arrival instructions. However, dispatchers may not follow this “all-caller protocol” script exactly; for example, dispatchers do not always ask consciousness and breathing questions [10]. Dispatchers are less likely to identify the need for T-CPR if they do not ask about consciousness and breathing, do not confirm that a patient’s breathing is *normal* [9, 11], or mistake agonal breathing sounds for normal respiration [6]. Furthermore, callers do not always provide clear or consistent answers to the all-caller protocol questions, which can also make identifying the need for T-CPR challenging [12].

There is some evidence that a dispatcher’s increased exposure to cardiac arrest calls can result in better outcomes for patients. Kuisma et al., found an association between the frequency of cardiac arrest calls a dispatcher handled and patient survival rates. For dispatchers who handled fewer than four such calls during their study period, survival to hospital discharge was 22 %; by contrast, when dispatchers took more than nine calls, survival was 39 % [13].

It is plausible that additional opportunities to practice cardiac arrest call-taking, with targeted training on evaluating the need for T-CPR, specifically, training on consistent querying of patient consciousness and breathing status, could improve dispatchers’ ability to identify suspected cardiac arrest and decrease time to start of T-CPR.^,^

The STAT-911 study seeks to provide 9-1-1 dispatchers with opportunities to practice and improve their medical emergency call querying skills through phone-based, standardized patient simulation training. The two primary study aims are to test if phone-based, standardized patient simulation training:

1. improves 9-1-1 dispatchers’ ability to identify the need for T-CPR during simulation calls.
2. reduces the time from call start to initiation of T-CPR during simulation calls.

The secondary aims are to:

1. test if participation improves 9-1-1 dispatchers’ ability to identify the need for T-CPR during actual cardiac arrest calls.
2. test if participation reduces the time from call start to initiation of T-CPR during actual cardiac arrest calls.
3. to evaluate if participation in the simulation training increases specific cardiac arrest identification querying skills during simulation calls.
4. test if participation improves dispatchers’ confidence in handling cardiac arrest calls.
5. estimate the cost of the simulation training as implemented in our study and estimate the projected costs for implementation by call centers.

## Conceptual framework

The STAT-911 simulation training seeks to improve dispatchers’ confidence and effectiveness in handling medical emergency calls through repeated practice, followed by immediate instructor feedback, in a risk-free simulated environment. Broadly defined, medical simulation is “the artificial representation of a phenomenon or activity that allows participants to experience a realistic situation without real-world risks” [14]. Simulation, when used effectively, can advance trainee communication skills [15, 16]. “Standardized patients” (live persons trained to portray patients with a variety of presenting complaints and pathologies) [17] are the standard for patient-provider communication simulations and a well-established and effective medical training method [18–20].

The STAT-911 training study incorporates essential elements of successful simulations, including: (a) the simulation is a valid representation of clinical practice, (b) immediate feedback, (c) repetitive practice, (d) increasing levels of difficulty and clinical variation, (e) a controlled environment, and (f) clearly defined outcome measures [17]. Post-simulation debriefing with feedback from an expert instructor can deepen the learning process, by guiding the trainees’ self-reflection, answering questions, and ensuring the application of training principles [21, 22].

While *in-person* simulation training with standardized patients has been used for over 40 years in medical education, there is a dearth of information on the effectiveness of using simulation training for phone-based health care systems such as 9-1-1 or nurse triage lines, even though these systems have a direct impact on patient safety, care, and health outcomes. The STAT-911 study will contribute evidence on the effectiveness and value of phone-based standardized patient simulation training to improve patient care.

## Methods and design

### Design

The STAT-911 study is a randomized controlled trial to evaluate the effectiveness of phone-based simulation training on improving 9-1-1 dispatchers’ ability to identify the need for T-CPR for patients in suspected cardiac arrest, and decreasing the time to start of T-CPR. Dispatchers from multiple 9-1-1 call centers will be randomized to an intervention or control group, and a post-intervention simulation test will be used to measure the primary outcomes. In addition to analyses of call processing behavior during the simulation sessions, we will obtain 9-1-1 call audio from all cardiac arrest incidents handled by dispatchers during their study period. OHCA incidents are identified by EMS and then linked to the 9-1-1 audio recording that matches the event.

**Fig. 1:**
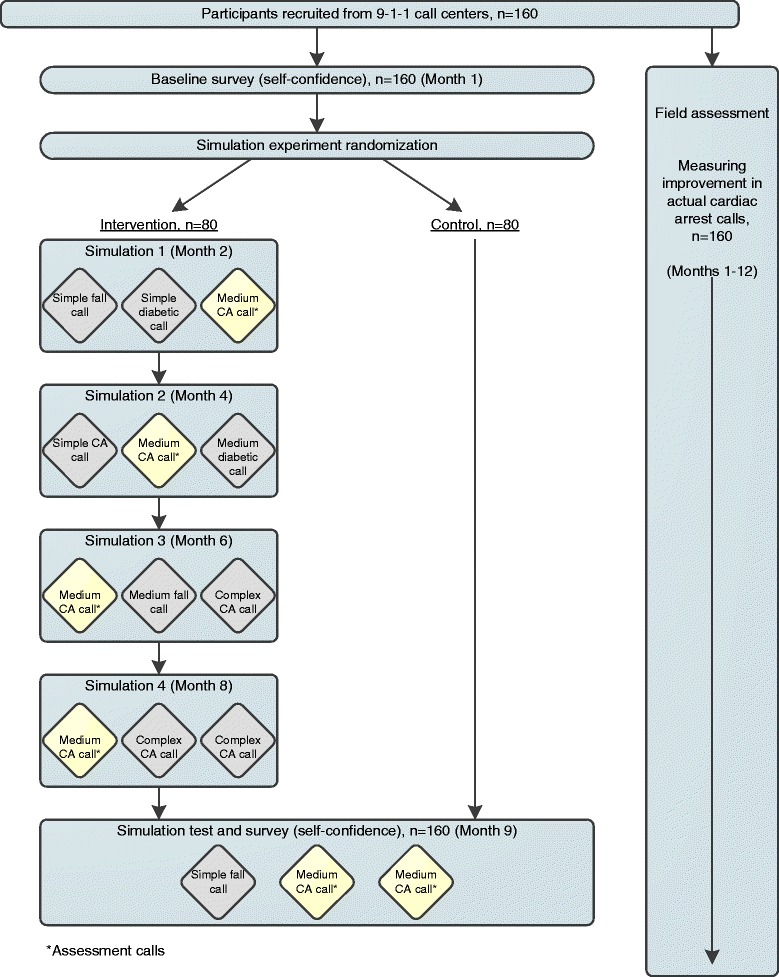
Study Design

The study design is shown in Fig. 1. The study is externally funded by the Agency for Healthcare Research and Quality, and the study protocol underwent peer-review by the funding body. The study was reviewed and approved by the University of Washington Human Subjects Division, #44640. The study adheres to the CONSORT guidelines.

### Setting

The study takes place in multiple 9-1-1 call centers in the western region of the United States. As each call center has slightly different medical emergency “all-caller” querying protocols; preferences regarding dispatcher recruitment activities and compensation for the trial; cardiac arrest incident surveillance; and procedures for data collection, we will secure individual Institutional Review Board (IRB) approval from the University of Washington for each call-center as they join the study.

### Study participants

After each 9-1-1 call center joins the study, we make repeated calls for dispatcher volunteers through flyers, email, and word-of-mouth. Upon enrollment, we obtain written informed consent from all dispatcher volunteers. After enrollment, dispatchers are randomized into either the intervention or control group. We anticipate being able to randomize 160 dispatchers into two groups (~80 per group), and obtain outcomes on at least 70 per group after loss to follow-up. We assume a loss to follow-up of about 12 % during the subjects’ one-year study period.

### The intervention

The intervention consists of four simulation training sessions conducted approximately every two months. Each of the four sessions includes three consecutive mock 9-1-1 calls of increasing complexity, followed by instructor-directed debriefing; a total of twelve simulated 9-1-1 training calls. The mock 9-1-1 calls feature “standardized callers” trained to act as bystanders calling 9-1-1 on behalf of a fictitious patient experiencing a medical emergency. Our “standardized callers” are members of the University of Washington Standardized Patient Program and are trained to follow scripts designed to simulate simple, medium, and more complex 9-1-1 caller scenarios. The scripts’ details are based on communication behaviors, observed in actual cardiac arrest calls to 9-1-1, that hinder efficient and accurate identification of cardiac arrest. For realism and skill-building variety, the call scenarios include an array of initial caller medical complaints; and varied patient medical histories, symptom presentations, and caller descriptions of consciousness and breathing status.

To structure our training, we identified four component parts in cardiac arrest call processing that, if handled skillfully, facilitate effective dispatch, identification of suspected cardiac arrest, and prompt pre-arrival T-CPR instructions: (1) managing the caller’s initial description of the medical emergency, (2) consciousness inquiry, (3) breathing inquiry, (4) starting T-CPR. The call scenarios, scripts, and instructor feedback are focused on teaching and reinforcing skills used to overcome common challenges in each call component: (1) Plausible, but ultimately inaccurate, initial descriptions of the medical emergency: call introduction *labels* (e.g., “my dad *fell*”); (2) ambiguous or inaccurate answers to the question “Is the patient *conscious*?” (e.g., “he’s sleeping”); (3) ambiguous or inaccurate answers to the question “Is the patient *breathing normally*?” (e.g., “he just snorts once in a while”); and (4) promptly initiating *T-CPR* instructions.

The call scenarios were vetted for realism and skill-building utility with Quality Improvement and training staff and the Dispatch Communication Leadership Group at King County EMS Division of Public Health: Seattle & King County (KCEMS-PHSKC). The simulation training was developed in consultation with the Institute for Simulations and Inter-professional Studies at the University of Washington.

### Procedures

At enrollment, study dispatchers complete a baseline survey that assesses confidence in handling difficult medical emergency calls (including cardiac arrest) and collects basic demographic and work experience information. Within each call center, dispatchers are stratified by duration of call center work experience and then, within those groups, randomized to the intervention arm or the control arm of the study. The Intervention group completes the four simulation training call sessions over a 6-8 month period. One month after the last simulation training call, all dispatchers (intervention and control) complete the simulation test: a call session in the same format as the simulation training sessions, consisting of three mock 9-1-1 calls, two of which are medium-complexity cardiac arrest calls. All dispatchers complete the follow-up survey that reassesses confidence in handling difficult calls. In addition, the intervention dispatchers also complete the simulation training experience survey, to evaluate their perceptions of the simulation experience; specifically, their attitude toward the training method, and its effect on their confidence and learning.

#### Simulation training

Simulation training call sessions take place at a workstation at the dispatcher’s call center or, in some cases, at a faithful recreation of a workstation at the dispatcher’s home. At a designated time, the instructor connects to the standardized caller, and then, in a recorded three-way call, to the dispatcher for each mock 9-1-1 call. The instructor fills out a feedback form while listening in to the three mock calls. After the third call ends, the instructor calls the dispatcher back and provides corrective or positive feedback on select call management skills used in the four call components (Label, Consciousness, Breathing, T-CPR), and later tested in the trained skills assessment. Outcome measures and data collection.

Table 1 outlines the outcome measures and data collection tools for each study aim. Further details on the outcome measures and data collection approaches follow.

**Table 1:** Outcome Measure and Data Collection Tools

Aim	Outcome measure	Data collection tool
Primary aims		
Test if phone-based standardized patient simulation training:		
1. Improves 9-1-1 dispatchers’ ability to identify the need for T-CPR during simulation calls.	Proportion of calls where dispatchers recognize the need for T-CPR, as indicated by the dispatcher starting T-CPR instructions in the simulation test.	Simulation test; *call event codes*
2. Reduces the time from call start to initiation of T-CPR during simulation calls.	The elapsed time (in seconds) from the start of the call to the start of T-CPR instructions in the simulation test.	Simulation test; *call event times*
Secondary aims		
Test if participation in the simulation training:		
1. Improves 9-1-1 dispatchers’ ability to identify the need for T-CPR during actual cardiac arrest calls.	Recognition of the need for T-CPR, as evidenced by the dispatcher starting instructions in actual 9-1-1 cardiac arrest calls.	Recordings of actual 9-1-1 cardiac arrest calls during the study period; call event codes
2. Reduces the time from call start to initiation of T-CPR during actual cardiac arrest calls.	The elapsed time (in seconds) from call start to initiation of T-CPR instructions in actual 9-1-1 cardiac arrest calls.	Recordings of actual 9-1-1 cardiac arrest calls during the study period; *call event times*
3. Increases specific cardiac arrest identification querying skills during simulation calls	Performance score on the eight trained skills in the simulation test.	Simulation test; *trained skills assessment*
4. Improves dispatcher’s self-confidence to handle cardiac arrest calls.	Self-reported confidence to handle cardiac arrest calls on a five point scale before and after the intervention.	Baseline survey & follow-up Survey
5. Estimate the cost of the simulation training as implemented and projected costs when implemented by call centers.	Calculated costs associated with intervention development and implementation; projected costs for field implementation.	Records for expenses: Study design; staffing time (instructor and standardized patients); dispatcher time

#### Coding of simulation and actual cardiac arrest calls

To assess training outcomes on call processing performance during simulation test calls and actual cardiac arrest calls, blinded study staff will code and time key call events in audio recordings of the simulated and actual cardiac arrest calls. The critical coded *call event* is if the dispatcher begins T-CPR instructions, which will confirm the dispatcher identified the need for T-CPR and measure if the training improves identification of the need for T-CPR. The critical recorded *call event time* is the elapsed seconds to the start of T-CPR, which will measure if the training reduces delays. In simulation test calls, staff will also complete a *trained skills assessment*, to score and measure performance on the eight trained call processing skills listed in Table 3.

For each recording study staff will follow the following procedures. First, study staff will code all conversations for occurrence of key *call events* (Table 2). Second, study staff will record the *call event times*, the elapsed time (to the nearest second) from the start of the call to when each key call event occurred. A coding and timing guide was written for consistent application of call abstraction procedures. To develop these guides, study staff coded and timed four call audio recordings to check consistency. These codes and times were compared, and coding rules were refined. The staff coded and timed an additional eight calls, and, when compared, were identical (Kappa statistic = 1). The correlation coefficient of the times to start of T-CPR instructions was 0.990. Table 2 outlines the call event codes.

**Table 2:** Call Event Codes and Times

Code	Interaction	Definition
C	First consciousness question	The first time the dispatcher asks a question that attempts to measure patient’s consciousness status.
B	First breathing question	The first time the dispatcher asks a question that attempts to measure patient’s breathing status.
TRAN	Transition from All-Caller questions to pre-arrival instructions	The point in the call where the dispatcher closes the all-caller interview and verbalizes the transition to T-CPR. This verbalization can be (1) the statement of the need for T-CPR, *or* (2) the start of actual T-CPR instructions.
INST	T-CPR instructions	The first time the dispatcher gives the actor a T-CPR instruction command.

For the *trained skills assessment*, study staff will code how dispatchers perform on each of eight training points using a detailed codebook developed to match each call’s script, training points and feedback. This codebook was tested with ten call audio recordings and refined in collaboration with the simulation training instructor. Staff engaged in three hours of training to familiarize themselves with the coding scheme and the codebook instructions. Then staff coded and conducted the trained skills assessment on eight calls. Coding and trained skills assessment results were identical (Kappa statistic = 1). The codebook defines how study staff is to complete the trained skills assessment form: Table 3.

**Table 3:** Trained Skills Assessment

Training Point Measurement	Result (yes/no/NA)
Does not get distracted by label at any point in the call	
Asks consciousness according to protocol script (Conscious/awake)	
Follows-up on first consciousness answer appropriately	
Closes consciousness inquiry after at most two unclear answers	
Asks breathing according to protocol script (breathing normally)	
Follows-up on first breathing answer appropriately	
Closes the breathing inquiry after at most two “not normal” breathing answers	
Transitions to appropriate pre-arrival instructions (T-CPR) promptly	

#### Power calculations

Initial sample size calculations called for 180 dispatchers. After collecting preliminary data, sample size calculations were repeated using updated parameter estimates of a baseline recognition rate of 70 %, a 20 percentage point improvement in recognition rate, a baseline median time to start T-CPR of 75 s, a 30 % reduction in the mean time to start T-CPR, and a conservative within individual correlation of 0.5 for both rate of recognition and time to start of CPR instructions. Under these assumptions we calculate we will need data on 140 dispatchers to have 80 % power of rejecting the null hypothesis of no change in the rate of recognition and 80 % power of rejecting the null hypothesis of no change in the time to start T-CPR, at the alpha = 0.05 level each. Power for the rate of recognition was calculated using the two-sample *t*-test given in the statistical analysis section of Lenth [23]. Power for time to start of T-CPR was calculated for simulation using the accelerated failure time analysis described in the statistical analysis section, but without adjustment for actor variation or script differences.

## Statistical analysis

### Primary aim analyses: differences in performance during the simulation test

The primary aim analyses will compare the rate of recognition of the need for T-CPR and the time to the start of T-CPR instructions between the intervention and control arms during the simulation test. The rate of recognition of the need for T-CPR will be compared between the intervention and control arms using a two sample *t*-test on the proportion of two T-CPR appropriate assessment calls for each individual (0, ½ or 1; each dispatcher completes two test calls).

The time to start of T-CPR is a potentially right-censored measurement; if the call receiver does not recognize the need for T-CPR prior to the end of the call, then the time until starting T-CPR is only known to be *at least* as long as the call duration (In practice, recognition of the need for CPR will occur in all cases when paramedics arrive on scene and attend to the patient). As the time to start of T-CPR must be analyzed using methods that can deal with censored observations, we will use an accelerated failure time model with log-logistic distribution to test the effect of randomization group. Standardized caller and call scenario will be included as covariates to allow for differences in standardized caller speaking speed and the complexity of the scripts, and allowing for a within subject correlation by using robust estimates of standard error.

### Secondary aims 1 & 2 analyses: effect of the intervention on actual cardiac arrest 9-1-1 calls

Dispatchers will have varying numbers of observations depending on how many actual cardiac arrest calls they receive during their study period. The recognition of the need for T-CPR will be analyzed using a random slopes mixed-effects logistic regression model, with 9-1-1 call center included as a covariate, and dispatcher as the random effect. The time to start of T-CPR will be analyzed using a parametric regression frailty model, with 9-1-1 call center included as a covariate, and dispatcher as the random effect.

### Secondary aim 3 analysis: differences in call processing skills during the simulation test

We will compare performance on the eight skill measures on the trained skills assessment between the intervention and control arms during the simulation test. The binary results for each of the eight skill measures (Table 3) will be compared across experiment arm (intervention v. control) using two-sample t-tests comparing the proportion of each skill (out of the two assessment calls) correctly demonstrated, if tested. Using the proportion as the outcome allows the denominator to vary between dispatcher, as not every skill is tested in every call. For example, if a dispatcher never conducts a consciousness inquiry, but first asks about breathing and then immediately starts T-CPR, then performance on the “follows-up on first consciousness answer appropriately” skill is not tested.

### Secondary aim 4 analysis: effect of the intervention on self-confidence

The effect of the intervention on change in self-confidence will be analyzed using a multivariate linear regression model with group and baseline self-confidence score as covariates and self-confidence score as reported on the follow-up survey as the outcome variable.

### Secondary aim 5 analysis: cost and cost-effectiveness

A cost of the intervention as implemented will be described with a focus on three types of costs: the design, the time of the instructor, and the time of the standardized patients and dispatchers. Data on the design costs are available from project financial records and can be spread across the trial participants and future participants if the intervention is adopted widely. The instructor is tracking his time on a random sample of days to estimate the ratio of time devoted to scheduling call sessions relative to time conducting, listening, and providing feedback during the call sessions. The time of the instructor, standardized patients and dispatchers is tracked during every training session and valued at average salaries for each profession. The cost analysis will also include projections of cost of the intervention when the trainings are conducted by supervisors at the call centers rather than the instructor on the project team.

An optional cost-effectiveness analysis may be conducted to estimate the cost per cardiac arrest patient who survives to hospital admission. We are working with the Public Health: Seattle & King County (PHSKC) Cardiac Arrest Surveillance System (CASS) with data on all cardiac arrest cases from 2001 to 2010 (*n* = 6357) to estimate parameters of a cost-effectiveness model. We plan to estimate the effect of bystander CPR on survival and costs of hospitalization. Preliminary estimates suggest that bystander CPR (T-CPR and unassisted) affects survival until hospital admission, but not other outcomes after hospitalization. If the same is true for hospital costs, we would focus on the cost per patient who survives to hospital admission, because survival and costs after hospital admission would be the same as other cardiac arrest patients.

## Discussion

Absent medical attention, chances for cardiac arrest survival decrease sharply with every minute after breathing stops. 9-1-1 dispatchers are often the first contact with a health care system for bystanders to cardiac arrest, and can improve health outcomes by quickly identifying suspected cases of cardiac arrest and promptly starting T-CPR. Previous research indicates that experience handling such calls may improve dispatcher recognition of cardiac arrest, but no studies have looked at a way to increase this experience through simulation. The STAT-911 study will evaluate the potential for using phone-based simulation training to increase dispatcher experience with cardiac arrest calls and improve their processing of medical emergency calls. It will also advance knowledge regarding best-practices in use of phone-based simulations for any health professionals who need to make treatment and triage decisions during phone-interactions with clients, patients, or bystanders. Although the use of “standardized patients” has been standard and effective in training health care providers in medical procedures and patient interaction, the use of “standardized callers” for training phone-operators in diagnosis, triage and treatment decisions is a novel application of this type of simulation training method and an exciting new area for research.
